# Is the Internal Rotation Lag Sign a Sensitive Test for Detecting Hip Abductor Tendon Ruptures after Total Hip Arthroplasty?

**DOI:** 10.1186/1754-9493-5-7

**Published:** 2011-04-17

**Authors:** Christian Ossendorf, Laurent Bohnert, Nadja Mamisch-Saupe, Daniel Rittirsch, Guido A Wanner, Hans-Peter Simmen, Claudio Dora, Clément ML Werner

**Affiliations:** 1 Department of Surgery, Division of Trauma Surgery, University Hospital Zurich, Raemistrasse 100, 8091 Zurich, Switzerland; 2 Department of Orthopaedics and Traumatology, Canton Hospital of Fribourg, Route de Bertigny, 1708 Fribourg, Switzerland; 3 Department of Diagnostic Radiology, University Hospital Zurich, Raemistrasse 100, 8091 Zurich, Switzerland; 4 Department of Orthopaedics, Balgrist University Hospital, University of Zurich, Forchstrasse 340, 8008 Zurich, Switzerland

## Abstract

**Introduction:**

Total hip arthroplasty (THA) is one of the most frequently performed procedures in orthopaedics and weakness of external rotators is often recognized thereafter. However, the etiology of lateral hip pain is multifaceted. For the diagnosis of abductor tendon rupture, magnetic resonance imaging (MRI) is the gold standard. As not every patient can be subjected to MRI, a clinical diagnostic test for easy detection of lesions of the abductor tendon is missing. Here, we present the internal rotation lack sign indicating abductor tendon pathology.

**Methods:**

The patient is placed in lateral position on a stretcher with hips and knees in neutral position. The knee is flexed to 45° and the hip passively abducted and elevated by the investigator. With the foot passively abducted, the patient is then asked to bring his knee in direction to the examination table. This motion is also tested passively. The test is regarded positive, if no internal rotation is possible and/or if this is painful. If groin pain is elicited during either of the exercises, the test is also rated positive.

**Results:**

We evaluated this test in 20 patients clinically and by magnetic resonance imaging (MRI). All patients demonstrated a positive internal rotation lag sign. Twelve of them lag of internal rotation and evidence of anterior abductor tendon rupture on MRI, 8 with lag of internal rotation and no evidence of abductor tendon rupture.

**Conclusion:**

The new clinical diagnostic sign presented here may improve the diagnosis of abductor tendon rupture in the future.

Level of Evidence: Diagnostic study, level I.

## Background

Total hip arthroplasty (THA) is one of the most frequently performed procedures in orthopaedic surgery done more than 300'000 times annually in the United States alone with an increase of 158% between 1990 and 2004 [1]. In addition, the number of total joint replacements is expected to rise to 600'000 by 2030 [2].

Among the regularly performed approaches to the hip joint are the anterior (Smith-Peterson) [3,4], anterolateral (Watson-Jones) [5,6], lateral transgluteal (Hardinge) [7], and posterior (Moore), [8] approach, virtually each of which has been modified for minimally invasive hip replacement surgery [9-14] and comprises its distinct features, problems and pitfalls [15-17]. In patients with THA implanted by a direct lateral transgluteal approach, weakness of external hip rotation due to iatrogenic damage to the external rotators is possible [18,19]. Heterotopic ossifications are a possible complication [20]. Among potential consequences are mechanical malfunction, muscular imbalance and hip pain.

Lateral hip pain, also referred to as 'trochanteric pain syndrome', frequently extends to the lateral thigh mimicking nerve root irritation or simulating lower back pain [21,22].

Lateral hip pain after total hip arthroplasty represents a frequent problem in orthopaedic outpatient departments. However, the exact cause is mostly unknown and delimitation to other pathologies is often difficult. Pertrochanteric pain syndrome may also hint to a rehabilitation deficit which is entirely solvable by physiotherapy. However, if not, this may hint to a lesion of the abductor tendon.

Today, in most centers specialized in joint replacement surgery with high case loads, the lateral transgluteal approach is merely used for revision surgery and for repair of the abductor tendon plate.

Disruptions of the abductor tendon are difficult to diagnose clinically as physical findings are sometimes subtle. Hence, magnetic resonance imaging (MRI) is an accurate tool for the diagnosis of tears of the gluteus medius and gluteus minimus tendons [23]. However, lack of physical correlation to MRI findings was reported [23]. Therefore, orthopaedic surgeons cannot be sure, whether they recognize distinct pathologies of the abductor tendon correctly if they attach too much importance to imaging or rely on MRI findings alone.

With internal rotation of the hip, the external rotators and their tendons get under tension, with pain indicating a lesion of the abductor tendon.

Here, we introduce the internal rotation lag sign of the hip for abductor tendon rupture which to our best knowledge has not been described or correlated to MRI findings yet. In addition, we briefly present our experience with the first 20 patients in which this diagnostic clinical test was used.

## Methods

All patients gave their informed consent to participate in the study. Patient rights are protected by local law that requires patient to be informed of the possibility of charge review for scientific purposes.

### Patient examination

**Figure 1:**
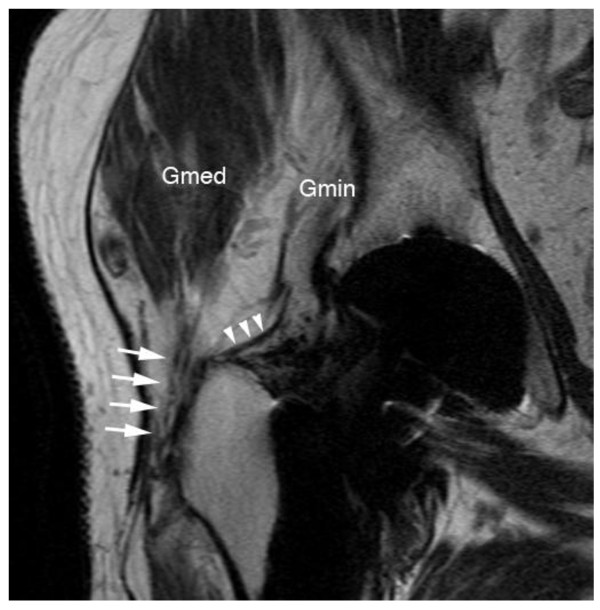
**clinical examination**: The patient is placed in lateral position on a stretcher with hips and knees in neutral position. The knee is flexed to 45° and the hip passively abducted with the leg elevated. Then the patient is asked to bring his knee in direction to the examination table. This motion is also tested passively. The test is regarded positive, if no internal rotation is possible and/or if this is painful. If groin pain is elicited during either of the exercises, the test is also rated positive.

The patient is placed in lateral position on a stretcher with hips and knees in neutral position. Hence, the knee of the tested side is flexed to 45° and the hip passively abducted and the leg passively elevated by the investigator. With the foot remaining passively abducted, the patient is asked to bring his knee in direction to the examination table. This motion is also tested passively. The test is regarded positive, if no internal rotation is possible and/or if this is painful. If groin pain is elicited during either of the exercises, the test is also regarded positive (Figure 1). Results are evaluated together with radiographs of the hip, to discriminate from patients with osteoarthritis of the hip or with loosening of the cup after THA.

Additionally, standard orthopaedic physical examination, including gate analysis (limping), range of motion, level of strength, and areas of localized tenderness is done.

### MR imaging

All MR imaging was performed on 1.5 Tesla MR imaging systems (Siemens Symphony, Espree or Avanto, Siemens Medical Solutions, Erlangen, Germany). A body matrix phased array coil and a spine array coil were used for all studies.

In all patients the following sequences were acquired: Intermediate-weighted fast spin-echo images in coronal plane [repetition time/echo time (TR/TE) 2590 ms/15 ms], field of view (FOV) 180 × 143 mm, NEX 1; matrix 512 × 256, echo train length (ETL) 7, section thickness 3 mm, a T1-weighted spin-echo sequence in transverse plane [TR/TE, 533 ms/12 ms], FOV 180 × 180 mm, NEX 1, matrix 512 × 256, section thickness 6 mm, as well as a sagittal T1-weighted sequence [TR/TE, 400 ms/12 ms], FOV 180 × 180 mm, NEX 1, matrix 384 × 269, section thickness 4 mm and a transverse short tau inversion recovery sequence (STIR) [TR/TE/TI, 4890 ms/45 ms/150 ms], FOV 180 × 180 mm, NEX 1, matrix 256 × 179, ETL 9, section thickness 7 mm.

## Results

We have evaluated the internal rotation lag sign in 20 patients (8 m, 12f; age 65 y (43-86)) treated in our outpatient department. All patients had the criteria of previous total hip arthroplasty and symptoms like pain or dysfunction of the hip joint, referred to as 'trochanteric pain syndrome'. Patients formed two groups: Group 1 (n = 12) with lag of internal rotation and evidence of anterior abductor tendon rupture on MRI; and group 2 (n = 8) with lag of internal rotation and no evidence of abductor tendon rupture. There were 7 men and 5 women in group 1, and 1 man and 7 women in group 2. Average age was 68 (43-86), and 60 years (43-75), respectively. Patient details are given in table 1. Mean follow-up of group 1 was 36 months (6-76), and 59 months (12-132) of group 2. Data acquisition and retrospective chart analysis were performed by an investigator independent of the surgical and outpatient clinic team. The institutional advisory board does not require its approval or informal consent for review of patients, records or images.

### Case Series

**Figure 2:**
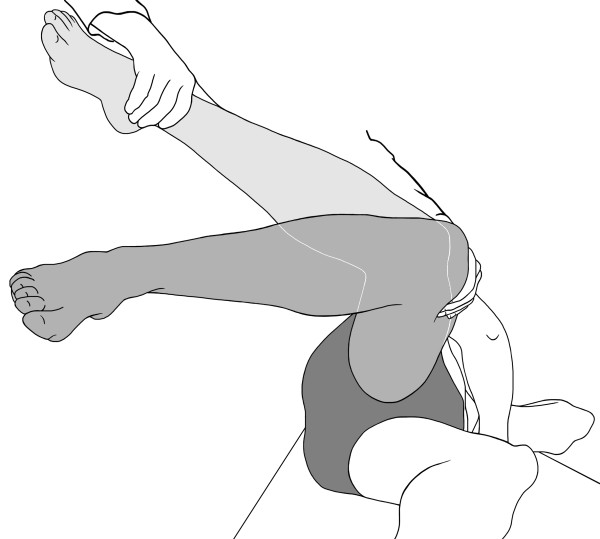
**lesion of the gluteus medius tendon muscle**: MRI T2-weighted turbo spin echo (TSE) coronal- MR image of an 80-year old male patient (TR/TE 4000/56 ms) shows a missing tendon attachment of the gluteus medius tendon at its antero-lateral tendon attachment at the greater trochanter (white arrows). Only some tendon fibers are depictable. Also note the thin tendon attachment of the gluteus minimus tendon (white arrowheads) and the fatty muscle infiltration of gluteus minimus muscle (Gmin). Only a slight fatty muscle infiltration of the gluteus medius muscle (Gmed) is shown.

All 20 patients demonstrated a positive internal rotation lag sign. In the 12 patients of group 1, all of which were operated on by a lateral, transgluteal approach, a rupture of the interior abductor tendon could be demonstrated on MRI (Figure 2). Of these, 2 patients had a partial rupture. In contrast, patients of a group 2 had insufficiency of the external rotators and lag of internal rotation, yet no rupture but merely insufficiency of the abductor tendon.

Four patients of group 1 required revision surgery due to rupture of the ventral gluteal tendon plate. Reoperations included THA, mobilization and revision/reconstruction of the ventral gluteal tendon plate (n = 3), tenotomy of the gluteal tendon, bursectomy, mobilization, debridement and refixation of the ventral gluteal tendon plate, soft tissue revision with or without rotation plastic using the vastus lateralis muscle, removal of wires after fracture of the trochanter, and its refixation. No revision surgery was necessary in patients of group 2.

## Discussion

Here, we describe a new clinical sign for inexpensive clinical diagnosis of abductor tendon rupture of the hip. Although not statistically confirmable, abductor tendon rupture appeared to be linked to the lateral, transgluteal approach in the present study. However, as the present study is merely descriptive and comprises a relatively small number of patients, throughout statistical testing including regression analysis, determination of sensivity, specificity, and predictive values was not possible. Further investigations with larger numbers of patients may be desirable.

Specific and supportive physical findings will aid the orthopaedic surgeon in making best use of multifaceted MRIs or to ease decision making, and to delimitate the multiple causes of trochanteric pain syndrome. Choosing the appropriate diagnostic tools based upon a sound clinical examination may result in higher precision of examinations and better clinical outcome.

Although the abduction mechanism can be repaired [24], damage to external hip rotators may be prevented by choosing an appropriate approach to the hip joint.

## Conclusion

The internal rotation lag sign may improve the diagnosis of abductor tendon rupture in the future, enhance and amend MRI findings, and potentially improve conclusiveness of clinical hip examination.

## List of abbreviations used

THA: total hip arthroplasty; MRI: magnetic resonance imaging; FOV: field of view; TR: repetition time; TE: echo time; ETL: echo train length; NEX: number of excitation; STIR: short tau inversion recovery; TSE: turbo spin echo; Gmin: gluteus minimus muscle; Gmed: gluteus medius muscle

## Competing interests

Each author certifies that he or she has no commercial associations (eg consultancies; stock ownership, equity interest, patent//licensing arrangements, etc) that might pose a conflict of interest in connection with the submitted article.

## Authors' contributions

CO, LB, CMLW designed the study. LB, CD, CMLW were involved in acquisition of the data. CO, LB, NMS, GAW, HPS, CD, CMLW analyzed the data. NMS, GAW, HPS, CD, CMLW contributed to interpretation of the data. CO, NMS, DR, GAW, HPS, CD, CMLW prepared the manuscript. DR, GAW, HPS, CD, CMLW contributed to revision of the manuscript. All authors read and approved the final manuscript.
